# Carney complex with PRKAR1A variant and breast/fibrolamellar carcinomas

**DOI:** 10.1038/s41439-026-00351-5

**Published:** 2026-05-28

**Authors:** Abílio Alonso Colares Perez, Laura Pinheiro Correia, Régis Ponte Conrado, Vinícius Nascimento Malheiro, Carlos Gustavo Hirth, Diane Isabelle Magno Cavalcante, Gustavo Rêgo Coelho, Ana Rosa Pinto Quidute, Danielle Calheiros Campelo Maia

**Affiliations:** 1 School of Medicine, Department of Clinical Medicine, Federal University of Ceará, Fortaleza, Ceará, Brazil; 2 Federal University of Ceará, Instituto do Câncer do Ceará Fortaleza, Fortaleza, Ceará, Brazil; 3 Department of Pathology and Legal Medicine, Federal University of Ceará, Fortaleza, Ceará, Brazil; 4 Department of Surgery, Walter Cantídio University Hospital, Federal University of Ceará, Fortaleza, Ceará, Brazil; 5 Division of Endocrinology, Walter Cantídio University Hospital, Federal University of Ceará, Fortaleza, Ceará, Brazil; 6 Department of Physiology and Pharmacology, Drug Research and Development Center (NPDM), Faculty of Medicine, Federal University of Ceará (UFC), Ceará, Brazil; 7 Department of Clinical Oncology, Walter Cantídio University Hospital, Federal University of Ceará, Fortaleza, Ceará, Brazil; 8 Present Address: Department of Pathology and Legal Medicine, Federal University of Ceará, Fortaleza, Ceará, Brazil

**Keywords:** Cancer genomics, Cancer genetics, Endocrine cancer, Cancer genetics, Tumour heterogeneity

## Abstract

Carney complex is a rare multiple neoplasia syndrome caused primarily by inactivating variants in the PRKAR1A gene, which encodes the regulatory subunit type I alpha of protein kinase A. Although classically associated with myxomas, lentigines, and endocrine tumors, the full tumor spectrum related to PRKAR1A loss of function is not completely defined. Here, we report a patient with a molecularly confirmed diagnosis of Carney complex carrying a de novo PRKAR1A (NM_002734.5):c.348+1G>A splice-site variant. In addition to typical manifestations, the patient developed two uncommon malignancies: invasive breast carcinoma (luminal B-like) at a young age and fibrolamellar hepatocellular carcinoma lacking the DNAJB1–PRKACA fusion typically found in sporadic cases. The coexistence of these two rare tumors in a single carrier of a pathogenic PRKAR1A variant expands the phenotypic variability associated with this condition and supports the possibility that PRKAR1A loss contributes to oncogenesis beyond the classical endocrine and mesenchymal tissues. This Data Report provides clinically relevant documentation of an unusual tumor presentation associated with a pathogenic PRKAR1A variant and underscores the importance of ongoing clinical surveillance in affected individuals.

Carney complex is an autosomal dominant multiple neoplasia syndrome characterized by spotty pigmentation, myxomas, and endocrine and non-endocrine tumors, most commonly caused by inactivating variants in *PRKAR1A*, which encodes the regulatory subunit type I alpha of protein kinase A (PKA)^1–3^. Loss of *PRKAR1A* function results in constitutive activation of the PKA signaling pathway and underlies the high penetrance and wide phenotypic variability observed in affected individuals^4,5^. Although the classical tumor spectrum is well established, recent observations suggest that *PRKAR1A* deficiency may predispose additional malignant phenotypes not traditionally associated with the syndrome^2,6–8^.

A 23-year-old Brazilian woman presented with facial lentigines and multiple blue nevi. Cardiac imaging revealed a 1.7 × 1.2 cm left atrial myxoma, which was surgically resected. Germline testing identified a de novo PRKAR1A (NM_002734.5):c.348+1G>A splice-site variant, confirming the diagnosis of Carney complex. According to ACMG/AMP guidelines, the variant was classified as pathogenic, supported by PVS1 (canonical ±1 splice-site loss of function), PS2 (de novo occurrence), PM2 (absence from population databases), and PP4 (highly specific phenotype).

The patient returned to medical follow-up at age 32 with a diagnosis of left-sided invasive breast carcinoma. She underwent bilateral adenomastectomy followed by chemotherapy, radiotherapy, and endocrine therapy with tamoxifen and goserelin. Breast carcinoma has recently been reported at increased frequency among women with Carney complex^6–8^, and somatic loss of the wild-type *PRKAR1A* allele has been demonstrated in several tumors, supporting a two-hit mechanism of tumorigenesis. Her tumor was estrogen-receptor positive and treated according to standard oncologic guidelines.

During oncologic staging, abdominal MRI revealed a hepatic lesion initially suspected to represent a hepatic adenoma. Percutaneous biopsy and subsequent extended right hepatectomy confirmed fibrolamellar hepatocellular carcinoma (FL-HCC). Gross examination of the resected specimen demonstrated a well-defined mass in the right hepatic lobe (Fig. 1). Histopathology showed lamellar fibrosis, intratumoral fibrous bands, and large polygonal tumor cells with abundant eosinophilic granular cytoplasm, consistent with FL-HCC (Fig. 2A–D). The tumor measured 9 × 8 cm, exhibited 60% necrosis, and showed no vascular or perineural invasion. In contrast to sporadic FL-HCC, typically driven by the DNAJB1–PRKACA fusion, Carney complex-associated FL-HCC demonstrates loss of PRKAR1A expression, as confirmed by immunohistochemistry for the PRKAR1A protein and fluorescence in situ hybridization-negative for DNAJB1–PRKACA fusion, supporting a biologically distinct mechanism of PKA pathway dysregulation independent of fusion-driven activation^2^.

**Fig. 1:**
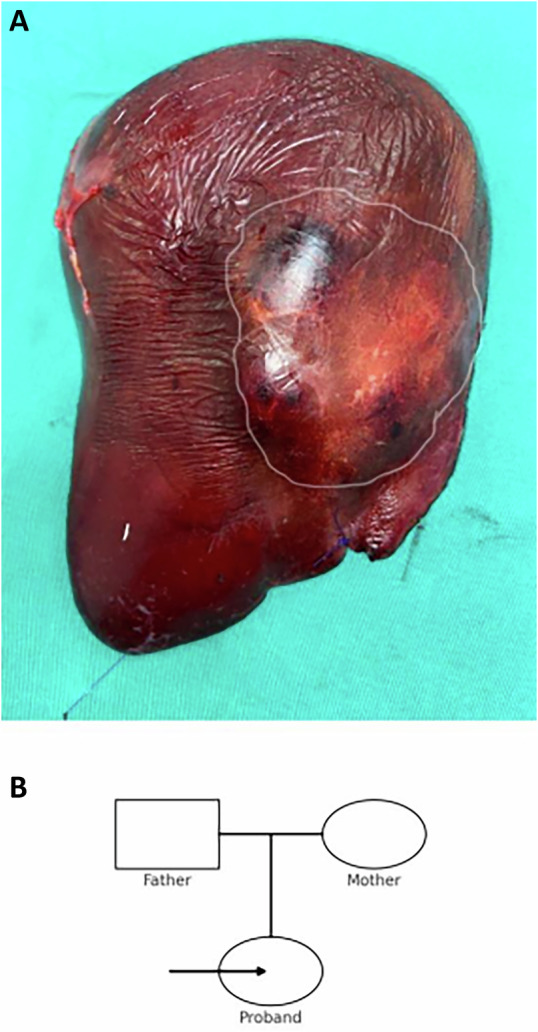
Gross appearance of the resected liver specimen. **A** Extended right hepatectomy specimen showing a well-defined mass corresponding to fibrolamellar hepatocellular carcinoma. **B** Pedigree showing the proband (arrow) carrying a de novo *PRKAR1A* pathogenic variant; both parents are clinically unaffected.

**Fig. 2:**
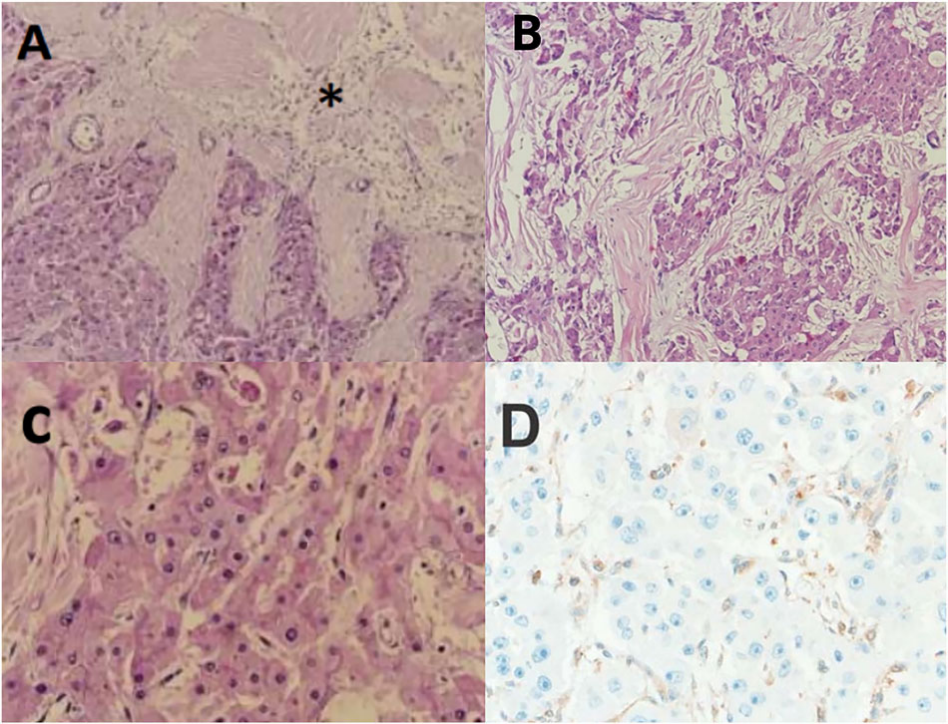
Histopathological features of fibrolamellar hepatocellular carcinoma. **A** Lamellar fibrosis and central fibrous region (40×)*.* **B** Additional representative field highlighting lamellar fibrosis and polygonal tumor cells (100×). **C** Neoplastic hepatocytes with abundant eosinophilic granular cytoplasm (400×). **D** PRKAR1A* expression was absent in the tumor cells of fibrolamellar carcinoma. Kupffer cells retained cytoplasmic expression and served as an internal control (400×). *PRKAR1A antibody (1:2,000; clone OTi6C7; Origene, MD, USA).

Evaluation for hypercortisolism revealed elevated urinary cortisol (363 µg/24 h; reference range 13–85 µg/24 h) and incomplete suppression on dexamethasone testing. However, the patient was receiving tamoxifen, a medication known to increase corticosteroid-binding globulin and to elevate total cortisol measurements, which can mimic biochemical hypercortisolism without true endogenous excess. Subsequent biochemical testing normalized after accounting for this interference. Adrenal imaging showed no nodules or morphological abnormalities. A pituitary MRI identified a non-functioning 8 × 4 × 4 mm microadenoma, a finding compatible with previously reported benign pituitary lesions in Carney complex^3^. Thyroid ultrasound revealed a 1.1 cm predominantly cystic nodule with benign features. A 9 mm spiculated pulmonary nodule noted on follow-up chest CT is under evaluation^9–14^.

The coexistence of early-onset breast carcinoma and FL-HCC in a young woman with a pathogenic *PRKAR1A* splice-site variant expands the recognized tumor spectrum of Carney complex. Both tumors arise in tissues where dysregulated PKA signaling may promote aberrant cellular proliferation. Although genotype–phenotype correlations in Carney complex are limited, splice-site variants such as c.348+1G>A typically result in complete loss of function and are associated with early multisystem involvement^4,5^. Additional genetic or epigenetic modifiers may influence tumor susceptibility, but these remain to be elucidated.

In summary, this report describes an unusual association of breast carcinoma and fusion-negative FL-HCC in a patient with a de novo *PRKAR1A* splice-site variant, contributing to the expanding phenotypic variability of Carney complex and supporting the importance of broad, long-term surveillance in affected individuals.

## hgv Database

The relevant data from this Data Report are hosted at the Human Genome Variation Database at 10.6084/m9.figshare.hgv.3657. The relevant variant information will be submitted to the Human Genome Variation Database as required and will be made available upon acceptance of the manuscript.
